# Research on Inherent Frequency and Vibration Characteristics of Sandwich Piezoelectric Ceramic Transducer

**DOI:** 10.3390/s22239431

**Published:** 2022-12-02

**Authors:** Yuren Lu, Chunguang Xu, Qinxue Pan, Quanpeng Yu, Dingguo Xiao

**Affiliations:** Key Laboratory of Fundamental Science for Advanced Machining, Beijing Institute of Technology, Beijing 100081, China

**Keywords:** piezoelectric ceramics, vibration model, inherent frequency, transducer vibration characteristics

## Abstract

Great progress has been made in the field of ultrasonic processing in recent years, and piezoelectric ceramic transducers have been widely used as drive sources. In this paper, a sandwich piezoelectric ceramic transducer is designed, and the vibration of each part of the transducer is analyzed by elastic mechanics and piezoelectric theory. According to its mechanical and electrical boundary conditions, the vibration model of the piezoelectric transducer was established. Based on the equivalent elastic modulus method for simplifying the pre-stressed bolts into a one-dimensional transducer vibration model, the relationship between the one-dimensional axial response frequency of the transducer and the length of each component was obtained. Based on the half wavelength theory, a transducer with the vibration node in the crystal stack and an inherent frequency of 15 kHz was designed and fabricated. In order to verify the natural frequency and vibration characteristics of the piezoelectric transducer, a laser vibration measurement system was built in this study. The vibration characteristics of the transducer under different parameters such as voltage and frequency were analyzed, and the accuracy of the vibration model was verified. The vibration states of the end surface of the transducer and the radial surface were evaluated at the first-order inherent frequency and second-order inherent frequency. The results show that the equivalent simplified model established in this study can effectively design the inherent frequency of the transducer, and the operation at the first-order inherent frequency meets the one-dimensional assumptions of this study. The transducer operating conditions measured in this study also provide a more detailed reference for ultrasonic processing applications.

## 1. Introduction

With the wide application of power ultrasound in advanced machining fields, sandwich piezoelectric transducers are valued and studied in ultrasonic milling [1,2], ultrasonic casting [3,4], and ultrasonic vibration for machining residual stress removal [5,6,7,8,9] because of their good electromechanical conversion characteristics. Although the structure and performance of piezoelectric ceramic transducers for different ultrasonic processing fields are not consistent, the inherent frequency of sandwich piezoelectric transducers is the most important performance parameter, which is the key to achieving high power output and high-efficiency energy conversion in practical applications. The excitation signal that deviates from the inherent frequency not only leads to the low efficiency of ultrasonic vibration but also to other vibration modes of the transducer, so it is very necessary to determine the inherent frequency before the application of the transducer.

Various methods have been used to study the relationship between the physical parameters of the PZT sandwich structure and its inherent frequency as well as other dynamic parameters. The most commonly used analytical method is the electromechanical equivalent model [10,11,12], whose transducer frequency characteristic equation can be obtained by drawing the mechanical-circuit 3-port or 4-port equivalent model according to mechanical boundary conditions. However, with the increasing demand for sound power of power transducers, the structure of multi-layer piezoelectric ceramic stacked pressure transducers is more popular, resulting in a very complex electromechanical equivalent model. In order to simplify the calculation model, the entire piezoelectric ceramic stack is regarded as an equivalent homogeneous body. The matrix transfer method [13,14,15,16] separates transducers into different independent elements to simulate multi-layer piezoelectric stack vibration, thus obtaining the vibration characteristics of piezoelectric ceramic stack transducers. Even more complex piezoelectric devices are still applicable under the assumption of one-dimensional wave propagation. Fu [17] proposed a hybrid transfer matrix approach for the modeling of the Rangzwan transducer, in which the prestressed bolt was modeled as a single quadrupole element, which was connected in parallel with other elements to establish a quadrupole element description of the piezoelectric Rangzwan transducer, thereby deriving the total transfer matrix and the inherent frequency of the transducer. Zhang [18] developed a simplified parametric analysis model of a piezoelectric transducer’s distribution that can effectively analyze piezoelectric transducers with thickness vibration modes. Finite element simulation is also a commonly used method to study the inherent frequency and vibration characteristics of the transducer. Abdullah, A [19] analyzed the mechanical behavior and inherent frequency of a sandwich transducer using the finite element method. Then, the equivalent circuit of the transducer was simulated to study the inherent frequency of the transducer that had been calculated [20]. Pérez-Sánchez [21] designed an ultrasonic transducer for acoustic cavitation using finite element analysis and found that when the ultrasonic transducer was used with a load, the frequency response of the transducer needed to be adjusted in terms of inherent frequency and voltage. Jiang, X [22] proposed an equivalent length method based on half-wavelength theory to analyze the inherent frequency of a sandwich transducer and analyzed the effect of different thicknesses of piezoelectric ceramics on the inherent frequency. Wei, X. [23] proposed a PSpice loss model for a sandwiched piezoelectric ultrasonic transducer undergoing longitudinal vibration using one-dimensional wave and transmission line theory to obtain the transducer’s resonance and anti-resonance frequencies. Chen [24] estimated the frequency of a sandwich transducer under various parameters by giant fuzzification-learning methods. Although the equivalent circuit method and matrix transfer method can model continuous systems with discrete components, which are valid around an inherent frequency, modeling the transducer’s continuous system vibration [25] can be valid in a wider range of frequencies. It can also describe the effect of geometry and material properties on the frequency response of the transducer. Based on the plane stress–strain assumption [26], many scholars have analyzed the vibrational characteristics of transducers. Piao [27] analyzed the inherent frequency and vibration modes of a transducer by solving the differential equations of piezoelectric motion. Other studies consider the coupling effect of radial and axial vibrations. The coupling vibration of a metal hollow cylinder is simplified to two equivalent one-dimensional vibrations by the apparent elasticity method [28,29,30,31]: one is the axial vibration of the equivalent cylinder and the other is the equivalent plane vibration in the radial direction. The inherent frequency equation of the metal hollow cylinder in the coupled vibration was derived, and the longitudinal and radial coupled resonant frequencies were calculated.

Although there are many studies and methods for analyzing the inherent frequency of a transducer, most of the current studies only focus on the operating frequency of the transducer, and the study of the operating state of the transducer near the inherent frequency is often neglected. At present, the evaluation of transducers’ performance is mainly described by the speed or amplitude of the front mass block—which is not only unintuitive but also incomplete with respect to the sandwich transducer—to evaluate the overall working state of the transducer. The operating conditions of the sandwich transducer lead to the observation of complex working conditions. Currently, the acoustic emission (AE) method [32,33], optical measurement method [34,35], mode degradation method [36], and other single-point methods are mainly used to measure the vibration amplitude of the transducer. Albareda [37] used a combination of optoelectronic methods, voltage and current measurements with an oscilloscope, and the vibration velocity of the transducer point with a laser vibrometer to evaluate a transducer’s operating state.

In this paper, the sandwich transducer is simplified into three parts: the front mass block, backing, and piezoelectric ceramic crystal stack, and the vibration model of the sandwich piezoelectric ceramic transducer is derived by continuous dielectric mechanics and a planar stress–strain assumption. A sandwich piezoelectric ceramic transducer with an inherent frequency of about 15 kHz is designed and fabricated by considering the mass effect of the compression bolt using the equivalent elastic modulus method. A doppler laser vibration measurement system was built, the vibration signal of the transducer was collected by the single-point method, and the inherent response frequency of the transducer was derived by FFT (Fast Fourier Transform). The operating state of the transducer under different parameters is discussed, the overall operating state of the transducer is swept and imaged, and the vibration state of the transducer in the axial and radial directions at the first-order and second-order resonant frequencies is measured.

## 2. Transducer Structure and Equivalent Models

**Figure 1:**
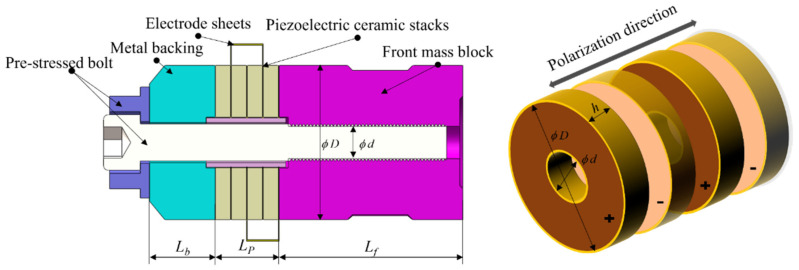
Schematic diagram of the structure of the sandwich piezoelectric transducer.

The sandwich transducer consists of front and back mass blocks, a piezoelectric ceramic stack consisting of piezoelectric ceramic circular pieces, pre-tensioning bolts, insulating sleeves, electrode sheets, etc. It is a continuous elastomer with a complex structure, as shown in Figure 1. The PZT-8 piezoelectric ceramic crystal stack is fixed between the front mass block and the metal backing by pre-stressed bolt connections.

**Figure 2:**
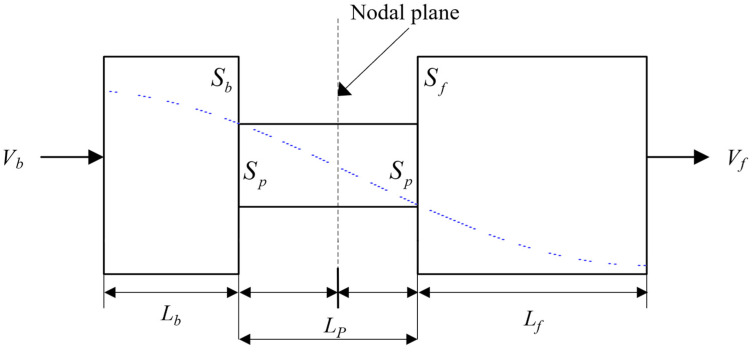
Schematic diagram of standing waves in a simplified sandwich transducer.

According to the wave propagation theory, the standing wave along the longitudinal axis of the transducer satisfies Equation (1) [22] during the resonant oscillation of the transducer:

(1) $$L=N\frac{\lambda }{2},$$

where L is the total length of the transducer; λ is the wavelength of the wave generated by the transducer’s vibration; N is a positive integer—when N = 1, the corresponding sandwich transducer becomes a half-wavelength transducer; and the total phase length of each component wave is π. As shown in Figure 2, both the metal backing and the end of the front mass block are at the peak of the displacement wave, where the vibration is most intense. The interface along the longitudinal axis where the wave propagation phase occurs is π/2 and the interface with zero displacement amplitude is the displacement node plane. The length of the metal backing is $L_{b}$, the cross-sectional area is *S_b_*, the vibration velocity is *V_b_*, the length of the front mass block is *L_f_*, the cross-sectional area is *S_f_*, the vibration velocity is *V_f_*, the length of the piezoelectric ceramic stack is *L_p_*, and the cross-sectional area is *S_p_*.

To satisfy the engineering requirements without affecting the working performance of the transducer, we need to make the following assumptions.

- The motion of the sandwich transducer along the axial direction is uniform, which means that the vibration caused by the piezoelectric ceramic is generated as a plane wave.
- It is assumed that the contact of the surface is perfect. Therefore, the reflection of the wave is neglected.
- Each ceramic piece in the piezoelectric ceramic stack is excited by a continuous sinusoidal wave, and the signal is in the same phase.
- The length of the transducer is more than three times its diameter, so the transverse Poisson effect is ignored and the transducer can be simplified to a one-dimensional model.
- The effect of the chamfer and thread of the transducer can be ignored; the transducer will be regarded as equal to the outside diameter.
- The acoustic impedance of air is much smaller than the transducer, so it is ignored. When the transducer is operating in the air, it is referred to as being under the “no-load” condition, and its stress is considered to be zero.
- The matched upper part is considered to be a solid cylinder with the center bolt integrated to simplify the boundary conditions.

## 3. Analysis Method of Transducer Inherent Frequency

### 3.1. Mechanical Vibration Equation of Piezoelectric Ceramic Stack

**Figure 3:**
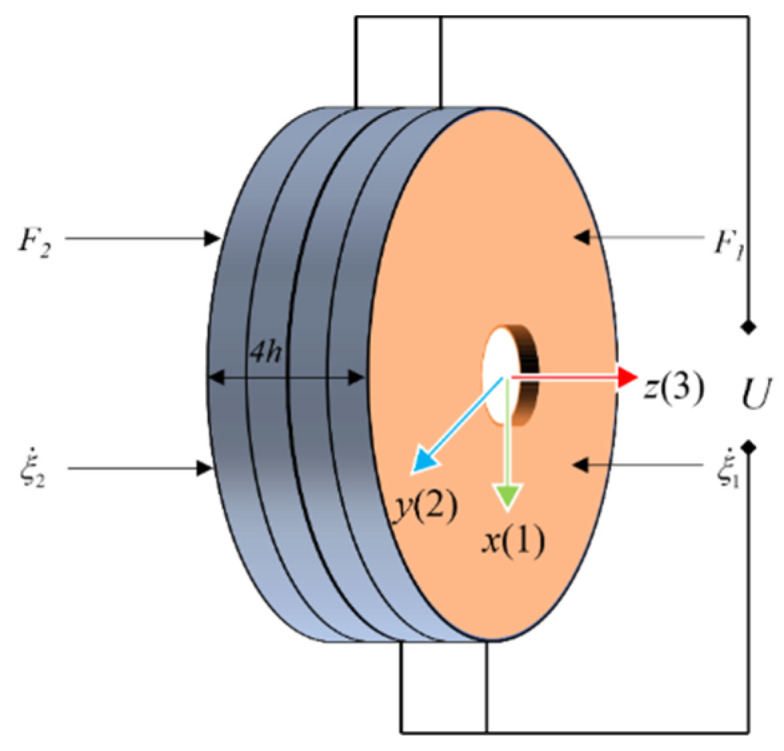
Electrical and mechanical boundary model of piezoelectric ceramic crystal stack.

The thickness vibration of a thin, circular piezoelectric ceramic has an axial polarization direction and the thickness of the piezoelectric ceramics is *h*. The mechanical and electrical connections of the piezoelectric ceramics are shown in Figure 3, and the stress and electric field conditions of the piezoelectric equation are shown in Equation (2):

(2) $$\{\begin{array}{c} T_{3}\neq 0,T_{1}=T_{2}=T_{12}=T_{13}=T_{23}=0\\ E_{3}\neq 0\\ \frac{\partial D_{3}}{\partial z}=0 \end{array},$$

where *T* is the stress on the piezoelectric ceramic and *E* is the electric field strength on the surface of the piezoelectric ceramic. Since this study simplifies the transducer into the one-dimensional axial continuous model, only the axial stress and electric field strength are considered. $T_{3}$ is the stress in the axial direction, $D_{3}$ is the electric displacement in the axial direction of the piezoelectric ceramic, and $E_{3}$ is the electric field strength applied to the piezoelectric ceramic in the axial direction.

When a voltage is applied on the surface of the piezoelectric ceramic, mechanical and electrical energy are converted into each other. Neglecting the transverse Poisson effect and assuming that only axial stresses exist, it can be concluded that $T_{3}\neq 0$. Since the axially applied voltage has $E_{3}\neq 0$, and considering that the ceramic is an insulating medium with no free charge in space, the potential shift vector is uniformly distributed and can be derived as $\partial D_{3}/\partial z=0$. The piezoelectric ceramic plates are polarized along the thickness direction, and every two adjacent piezoelectric ceramic plates are polarized in opposite directions so that the vibration can be better transmitted and superimposed.

According to the above boundary conditions, the piezoelectric matrix is shown in Equation (3):

(3) $$\left[\begin{array}{l} S_{3}\\ E_{3} \end{array}\right]=\left[\begin{array}{cc} s_{33}^{D} & -g_{33}\\ -g_{33} & \beta^{T}{}_{33} \end{array}\right]\left[\begin{array}{l} T_{3}\\ D_{3} \end{array}\right],$$

where $S_{3}$ is the strain in the axial direction, $s_{33}^{D}$ denotes the elastic constants of the piezoelectric ceramic at the equipotential boundary condition, $g_{33}$ is the piezoelectric constant, and $\beta^{T}{}_{33}$ is the dielectric isolation rate of the piezoelectric ceramic.

From the partial differential equation of motion, it follows that:

(4) $$\rho_{p}\frac{\partial^{2}\zeta (z,t)}{\partial t^{2}}=E_{p3}\frac{\partial \zeta (z,t)}{\partial z},$$

where $\rho_{p}$ is the density of the piezoelectric ceramic, $E_{p3}$ is the equivalent modulus of elasticity in the axial direction of the piezoelectric ceramic, and ζ(z,t) is the longitudinal displacement of the cross-section.

Merging Equation (4) into the piezoelectric matrix yields:

(5) $$\rho \frac{\partial^{2}\zeta (z,t)}{\partial t^{2}}=\frac{\partial T_{3}(z,t)}{\partial z}-\frac{g_{33}}{s_{33}^{D}}\frac{\partial D_{3}(z,t)}{\partial z},$$

The boundary condition, Equation (5), can be simplified as:

(6) $$\frac{\partial^{2}\zeta (z,t)}{\partial t^{2}}=\nu^{2}\frac{\partial^{2}\zeta (z,t)}{\partial z^{2}},$$

where $\nu =\sqrt{\frac{1}{\rho s_{33}^{D}}}$ is the propagation velocity of the one-dimensional longitudinal vibration in the piezoelectric ceramic crystal stack.

Since a continuous sinusoidal signal is applied to the piezoelectric ceramic, the vibrational response of the transducer is also a simple harmonic:

(7) $$\zeta (z,t)=\zeta (z)\cdot e^{jwt}=(m\sin kz+n\cos kz)\cdot e^{jwt},$$

where *m* and *n* are coefficients to be determined, which can be determined by the boundary conditions of the piezoelectric ceramics.

The displacement distribution in the axial direction of the piezoelectric ceramic crystal stack can be derived as:

(8) $$\{\begin{array}{c} \zeta (z)=\frac{\zeta_{1}\sin k(l-z)+\zeta_{2}\sin kz}{\sin kl}\\ \zeta_{1}=\zeta {|}_{z=0}\\ \zeta_{2}=\zeta {|}_{z=l} \end{array},$$

where $\zeta_{1},\zeta_{2}$ denote the vibration displacement of the two ends of the piezoelectric ceramic, so according to the mechanical boundary conditions $F_{1}=-ST_{3}{|}_{z=0},F_{2}=-ST_{3}{|}_{z=l}$, the mechanical vibration equation can be obtained as:

(9) $$\{\begin{array}{c} F_{1}=[j\rho vS\tan\frac{kh}{2}+(\frac{\rho vS}{j\sin kh}-\frac{n^{2}}{j\omega c_{0}})]{\dot{\zeta }}_{1}+(\frac{\rho vS}{j\sin kh}-\frac{n^{2}}{j\omega c_{0}})]{\dot{\zeta }}_{2}+nV\\ F_{2}=(\frac{\rho vS}{j\sin kh}-\frac{n^{2}}{j\omega c_{0}})]{\dot{\zeta }}_{1}+[j\rho vS\tan\frac{kh}{2}+(\frac{\rho vS}{j\sin kh}-\frac{n^{2}}{j\omega c_{0}})]{\dot{\zeta }}_{2}+nV \end{array},$$

### 3.2. Vibration Model of Transducer

**Figure 4:**
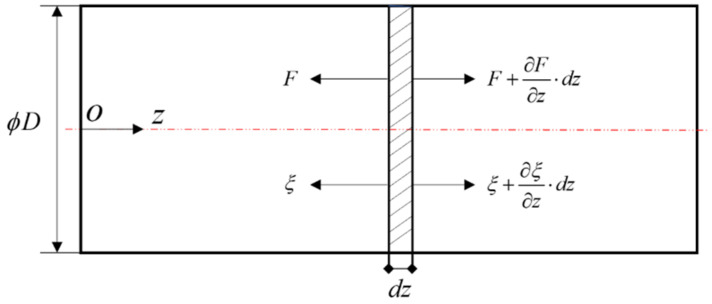
Simplified model of transducer vibration.

For this system, the parts of the transducer are all columns with the same cross-section, and assuming that the cross-sectional rods are composed of uniform, isotropic materials and neglecting their mechanical losses, the stress distribution on the rods’ cross-section is considered to be uniform. Figure 4 represents the forces and displacements of the unit body at any interface of the equal diameter sandwich transducer, which can be expressed as:

(10) $$dF=E\frac{\partial }{\partial z}(\frac{\pi D^{2}}{4}\frac{\partial \zeta (z,t)}{\partial z})dz=\rho \frac{\pi D^{2}}{4}dz\frac{\partial^{2}\zeta (z,t)}{\partial t^{2}},$$

Combinning Equation (8) into Equation (10) can lead to:

(11) $$\frac{\partial^{2}\zeta (z,t)}{\partial z^{2}}=\frac{\rho }{E}\zeta (z,t),$$

Since the longitudinal vibration of the multilayer piezoelectric ceramic sandwich transducer generally exists at the nodal plane of the piezoelectric ceramic element part, it is divided into four parts, as shown in Figure 2, where the subscript 𝑛 denotes each section of the sandwich transducer: 𝑛 = 1, 2, 3, and 4. The vibration equation of each section is a simple harmonic vibration, so the vibration speed equation of each section can be expressed as:

(12) $$\frac{\partial^{2}v_{n}(z)}{\partial z_{n}{}^{2}}+k_{n}{}^{2}v_{n}(z)=0,$$

The vibration speed and force on the mass block part can be expressed as:

(13) $$\left\{ \begin{array}{l} v_{n}(z_{n})=A_{n}\sin(k_{n}z_{n})+B_{n}\cos(k_{n}z_{n})\\ F_{n}(z_{n})=\frac{E\pi D^{2}}{j4\omega }\cdot \frac{\partial v_{n}}{\partial z_{n}}=-jZ_{n}[A_{n}\cos(k_{n}z_{n})-B_{n}\sin(k_{n}z_{n})] \end{array}\right.,$$

where $Z_{n}$ is the acoustic impedance of each part of the transducer and $A_{n}$, $B_{n}$ are the coefficients to be determined by the boundary conditions.

The vibration node’s cross-section is used to analyze both sides separately, and the boundary conditions are shown in Table 1.

The frequency equation describing the nodal plane can be obtained by bringing the boundary conditions in Table 1 into Equation (13).

(14) $$\left\{ \begin{array}{l} \cot(k_{1}L_{1})=\frac{z_{2}}{z_{1}}(\tan(k_{2}L_{2})-j\frac{z_{f}}{z_{2}})\\ \tan(k_{3}L_{3})\cdot \tan(k_{4}L_{4})=\frac{z_{3}}{z_{4}} \end{array}\right.,$$

If the input impedance $z_{f}$ is neglected, the frequency equation for the left and right sides of the transducer vibration node can be simplified as:

(15) $$\left\{ \begin{array}{l} \tan(k_{1}L_{1})\cdot \tan(k_{2}L_{2})=\frac{z_{1}}{z_{2}}\\ \tan(k_{3}L_{3})\cdot \tan(k_{4}L_{4})=\frac{z_{3}}{z_{4}} \end{array}\right.,$$

### 3.3. Equivalent Elastic Modulus Method of Transducer

The piezoelectric ceramic group of the longitudinal vibration sandwich transducer consists of four PZT-8 piezoelectric ceramics and four copper electrode sheets bonded by epoxy resin. The front and rear mass blocks of the transducer are made of a Duraluminum alloy, and the nuts and preload bolts are made of C45E4 steel; the properties of the materials are shown in Table 2 [38].

As can be seen from Figure 1, the transducer is not composed of the same material within the same cross-section, so the equivalent elastic modulus method is used to correct the material parameters in this paper. Assuming that the velocity and displacement of each cross-section of the pre-stressed bolt are consistent with the external piezoelectric ceramic wafer, then the equivalent density *ρ*′, equivalent sound velocity *c*′, and equivalent cross-sectional area *s*′ of the piezoelectric ceramic and pre-tensioned bolt can be chosen to replace the original ceramic acoustic parameters:

(16) $$\left\{ \begin{array}{l} \rho^{\prime }=\frac{\rho_{1}s_{1}+\rho_{2}s_{2}}{s^{\prime }}\\ c^{\prime }=\frac{c_{1}s_{1}+c_{2}s_{2}}{s^{\prime }}\\ Z^{\prime }=\rho^{\prime }c^{\prime }s^{\prime }\\ s^{\prime }=s_{1}+s_{2}\\ k^{\prime }=\frac{2\pi f}{c^{\prime }} \end{array}\right.,$$

where $\rho_{1}$, $s_{1}$, $c_{1}$ are the density, cross-sectional area, and sound velocity of PZT-8, respectively. $\rho_{2}$, $s_{2}$, and $c_{2}$ are the density, cross-sectional area, and sound velocity of the pre-stressed bolt. $Z^{\prime }$ and $k^{\prime }$ and are the equivalent impedance and equivalent wave number. Structural equivalence means that the structure of different materials in the same section of the vibration direction is equal to the structure of a single material, and the material property parameters of the equivalent structure are shown in Table 3.

## 4. Experimental Methods

### 4.1. Design and Inherent Frequency Measurement of Piezoelectric Transducer

The propagation speeds of the acoustic waves in the three components of the transducer are different. Therefore, Equation (1) cannot be used to directly calculate the structural parameters of each part of the actuator, but the propagation frequencies of each part of the transducer are the same:

(17) $$\{\begin{array}{c} \frac{\theta_{b}}{L_{b}}=\frac{2\pi }{\lambda_{b}},\frac{\theta_{f}}{L_{f}}=\frac{2\pi }{\lambda_{f}},\frac{\theta_{p}}{L_{p}}=\frac{2\pi }{\lambda_{p}}\\ L_{b}+L_{f}+L_{p}=L\\ \begin{array}{l} \theta_{b}+\theta_{f}+\theta_{p}=\pi \\ \lambda =f\cdot c_{\left(b,f,p\right)} \end{array} \end{array},$$

where $c_{b}$, $c_{p}$, and $c_{f}$ represent the propagation velocities in the backing, the piezoelectric ceramic, and the front mass block, respectively. $\lambda_{b}$, $\lambda_{p}$, and $\lambda_{f}$ represent the wavelengths in the corresponding components, respectively. As shown in Figure 2, the lengths of the backing, the piezoelectric ceramic stack, and the front mass block are $L_{b}$, $L_{P}$, and $L_{f}$, respectively, and the phase lengths of each element are $\theta_{b}$, $\theta_{p}$, and $\theta_{f}$, respectively.

**Figure 5:**
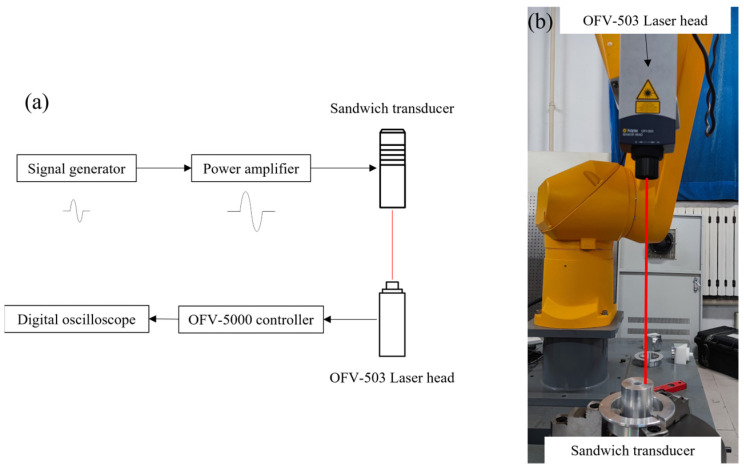
Single-point transducer vibration signal acquisition device: (**a**) Schematic diagram of transducer free vibration signal acquisition system; (**b**) Free vibration signal acquisition system of transducer.

In order to verify the accuracy of calculating the inherent frequency of the one-dimensional simplified sandwich transducer, the test system is composed of a polytec doppler laser vibrometer (controller model OFV-5000; laser head model OFV-503), Power ultrasonic transducer, high-power supply (Yongke, Ningbo, China), signal-generating device (RIGOL-DG1022U, Suzhou, China), digital oscilloscope-MDO3102 (Tektronix, U.S.), and vibration isolation test bench, as shown in Figure 5. The pulse signal is generated by the signal-generating device to enable the transducer to produce free attenuation vibration, and the vibration velocity of the end face of the transducer is measured by the laser vibrometer. After the vibration signal is converted into a voltage signal, it is obtained on the digital oscilloscope and analyzed by FFT to obtain the inherent frequency of the transducer.

### 4.2. Measurement of Transducer Working State

In order to accurately evaluate the working state of the transducer, the surface vibration energy of the transducer is used as the evaluation index in this paper [31].

(18) $$I=\frac{1}{2}\rho cv^{2},$$

**Figure 6:**
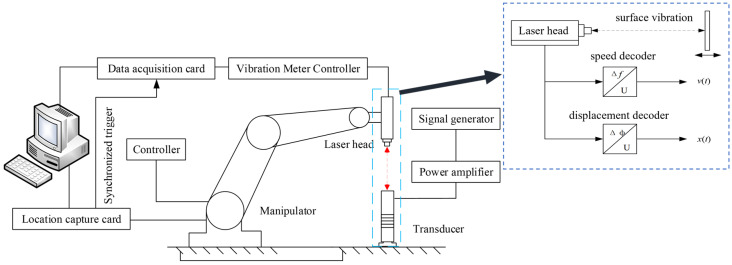
Working state measurement system of sandwich transducer.

Previous studies have only used laser vibrometry and acoustic emission methods to detect the vibration amplitude or vibration velocity of a single point of a piezoelectric sheet or sandwich transducer, which only enables the acquirement of the vibration state of a single point of the transducer and does not characterize the overall surface at the end of the transducer, so the characterization of the overall working state of the transducer is not complete. Therefore, this paper adopts a manipulator-based sandwich transducer sound intensity measurement system, as shown in Figure 6, which consists of a six-degree-of-freedom manipulator with a laser vibrometer that sweeps the working surface of the transducer along a preset trajectory, collecting the coordinates of the manipulator’s position and surface vibrational data through a position acquisition card and a data acquisition card, wherein the position acquisition card triggers the data acquisition card to achieve synchronous acquisition and uploads the data to the host computer software via a PCI bus. The above computer software processes the data in real time, displays the vibration information in A-sweep, extracts its eigenvalues, matches them with the positional coordinates one by one, and displays the sound field distribution in C-sweep. The robot adopted was a six-degree-of-freedom Staubli RX160 robot with a repeatability of 0.05 mm. The selected laser vibrometer was Germany Polytec laser interferometer, including the controller OFV-5000 and the laser head OFV-503. The data acquisition card was M2i.3120 from Spectrum, Germany, with a frequency up to 10 MHz. The signal generator was DG1022U dual-channel arbitrary waveform generator from Puyuan. Power amplifier was the signal source small excitation signal employing the linear method and impedance matching to achieve high-power excitation of the power transducer.

## 5. Results and Discussion

### 5.1. Transducer Inherent Frequency Analysis

The length of each part of the transducer is set as $L_{P}=L_{1}+L_{3}=41\;\mathrm{mm}$, *L*_2_ = 74 mm, and *L*_4_ = 32 mm; the outer diameter of the piezoelectric ceramic $\phi D_{r}=60\;\mathrm{mm}$; the inner diameter $\phi d_{r}=30\;\mathrm{mm}$; the thickness is *h* = 10 mm; and the thickness of the electrode sheet is 0.25 mm. Therefore, the piezoelectric ceramic’s inherent frequency of 15.15 kHz can be obtained from Equations (15) and (17), where *L*_1_ = 3 mm and *L*_3_ = 38 mm, and the node’s cross section is located in the piezoelectric ceramic stack and near the front mass block.

**Figure 7:**
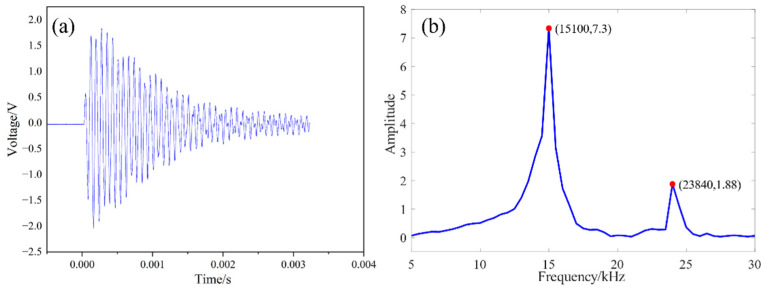
Free attenuation signal and spectrum analysis of high power piezoelectric transducer: (**a**) Free vibration signal of transducer; (**b**) FFT diagram of vibration signal.

The free vibration signal of the transducer after being excited by the pulse is observed using a digital oscilloscope, as shown in Figure 7a. The obtained spectrum is shown in Figure 7b. There is only one peak in the analysis’s frequency range, and the corresponding frequency value is 15.10 kHz, which is a 0.05 kHz difference from the design. The inherent frequency of the transducer is 15.44 kHz when ignoring the pressing bolt without an equivalent calculation of each part of the transducer.

### 5.2. Working Performance Measurement and Analysis of Sandwich Piezoelectric Transducer

The working performance of the sandwich transducer will directly affect the operational performance of the power ultrasonic system. In order to maintain the best performance of the transducer in its working state, it is necessary to analyze the factors affecting the working performance of the transducer. The influence of the excitation signal on the transducer is considered in this study. The characteristics of the excitation signal include voltage, frequency, and continuity.

**Figure 8:**
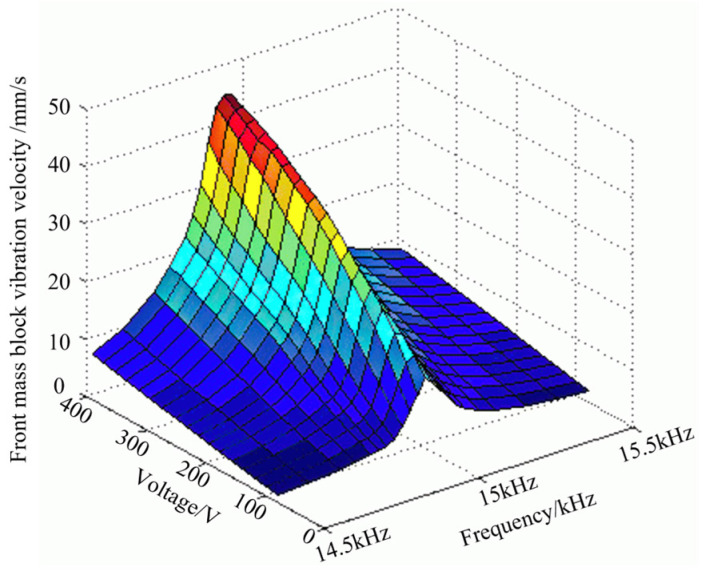
Performance cloud diagram of sandwich transducer.

Combined with the working range of the piezoelectrically powered transducer, the voltage range of the excitation signal is selected to be 80~400 V. The excitation signal is selected near the first-order inherent frequency, and the frequency range is 14.5~15.5 kHz. The signal generator outputs a continuous sine wave, and the laser vibrometer is used to detect the vibration velocity of the end face of the front mass block. The measurement results are read on the digital oscilloscope and the performance cloud diagram of the piezoelectric transducer is obtained after fitting, as shown in Figure 8. It can be seen that as the excitation voltage increases, the vibration velocity of the transducer also increases, and the closer the excitation frequency is to the inherent frequency, the higher the vibration velocity at the end of the transducer. However, with the increase in the excitation voltage, the optimal working frequency of the transducer is not fixed, and the vibration velocity at the end of the transducer is not linear with respect to the excitation voltage.

**Figure 9:**
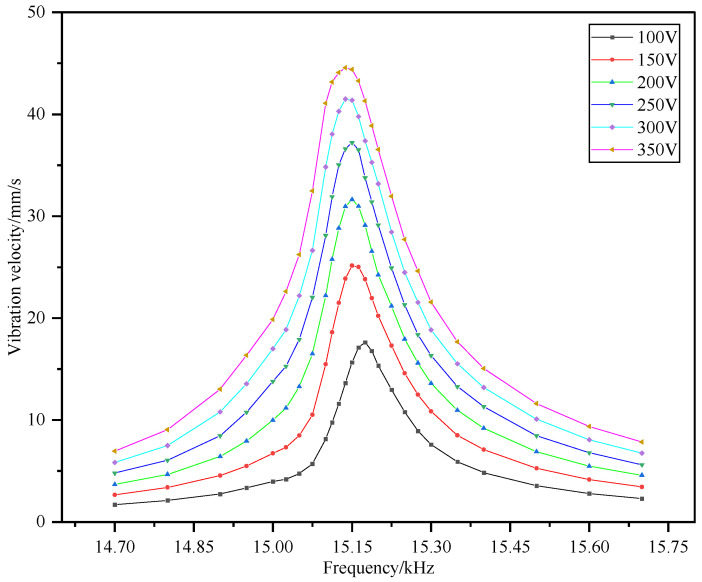
Power transducer frequency characteristic curve.

In order to analyze the influence of the excitation frequency on the working characteristics of the sandwich transducer, six groups of constant voltage excitation were selected to obtain vibration velocity curves with different frequencies, as shown in Figure 9.

Corresponding to each voltage value, there is a peak in the frequency range, and the transducer deviates from the inherent frequency of the vibration, which decreases significantly. This shows that if the power transducer does not work at the optimal frequency, its working efficiency is greatly reduced. At the same time, the shape of the curve is similar to the peak at the resonant frequency shown in Ref [25], and the experimental results are consistent with the theory. In addition, it can be found that the resonant frequency of the transducer is different under different excitation voltages. For example, the resonant frequency of the transducer is 15.16 kHz when the excitation voltage is 100 V, and the resonant frequency is 15.15 kHz when the excitation voltage is 150 V and 200 V. As the excitation voltage continues to increase, the change in the resonant frequency is no longer obvious—specifically, it is 15.14 kHz. In general, the change in the resonant frequency is not obvious. In practical applications, the maximum working efficiency can be obtained by fine-tuning the frequency according to the excitation voltage.

Based on the continuous excitation method, the inherent frequency obtained by analyzing the above data is about 15.15 kHz. Based on the electromechanical equivalent circuit method proposed in Ref [25], the inherent frequency of the transducer is 15.25 kHz. The difference in the inherent frequency obtained after analysis is due to the machining assembly’s accuracy and preload. The inherent frequency based on the pulse excitation method is 15.10 kHz. This can be explained by the different excitation modes of the pulse wave and the continuous wave of the transducer, which lead to the difference in the mechanical vibration state of the transducer.

**Figure 10:**
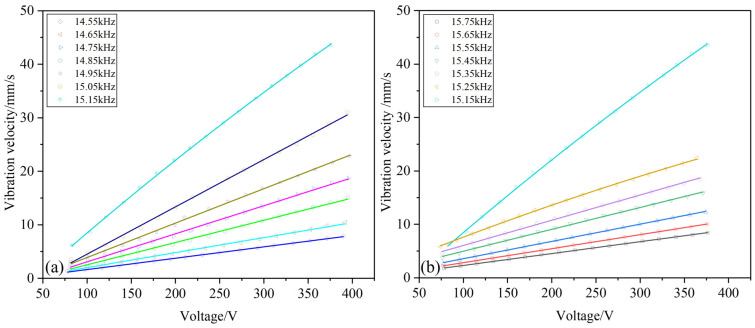
Voltage response curve of frequency for piezoelectric transducer near 15.15 kHz: (**a**) diagram of voltage-frequency relationship in 14.55–15.15 kHz frequency, (**b**) diagram of voltage-frequency relationship in 15.15–15.75 kHz frequency.

Since the inherent frequency of the transducer is 15.15 kHz, several sets of frequencies near the two sides of the frequency were selected, and the voltage characteristic curve is shown in Figure 10.

It can be found that at a frequency far from the inherent frequency, the change in the vibration velocity with the voltage is basically linear, but the closer to the inherent frequency, the weaker the linear relationship. When the vibration frequency is 15.15 kHz, a nonlinear relationship between the vibration velocity and the voltage can be seen, indicating that when the power transducer is close to the inherent frequency, the vibration amplitude changes dramatically; consequently, the bolt preload is not sufficient to offset the stress generated by the large displacement vibration, resulting in a gap between the transducer structures, thereby blocking the propagation of vibrations, and, finally, causing the measured velocity to differ from the theoretical value.

### 5.3. Measurement of Working State of Sandwich Transducer

**Figure 11:**
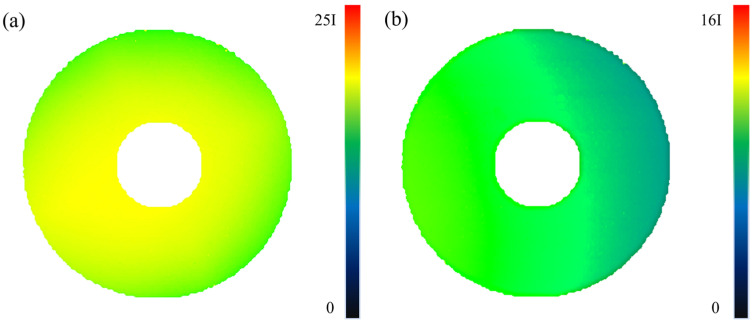
Radiation intensity distribution of sandwich transducer end face: (**a**) vibration intensity cloud diagram of transducer end under first order natural frequency; (**b**) Vibration intensity cloud diagram of transducer end under second order natural frequency.

The sandwich transducer uses the vibration of the piezoelectric ceramic along the thickness direction to realize its overall vibration along the axis. Under ideal conditions, the piezoelectric ceramic is in a uniform vibration state. However, the sandwich transducer relies on the pre-tightening of the bolts; the vibration amplitude and phase of the particles in the end face are different along the radial direction, which will affect the application of the power transducer system. Since the sandwich transducer is a vibration system, its vibration mode is related to the excitation frequency, and the function of the sandwich transducer is to use the longitudinal vibration to transfer energy; thus, it is necessary to find the frequency corresponding to the longitudinal vibration. It can be seen that the first-order and second-order inherent frequencies of the transducer are 15.10 kHz and 23.84 kHz. The vibrational intensity distribution of the transducer’s surface obtained by continuous signal excitation is shown in Figure 11. Since the pre-stressed bolt of the transducer runs through the whole transducer, the working area of the face is annular.

In Figure 11, I is the sound intensity value of a unit, which is 7 W/m^2^*,* and the corresponding vibration velocity is 1 mm/s. Comparing the first-order and second-order radiation intensity distribution maps, it is clear that the second-order radiation intensity of the end face of the transducer is almost reduced to one-quarter of the first-order. According to the sound intensity calculation formula, the particle vibration velocity of the end face is reduced to about one-half. Through Figure 7b, it can also be seen that during vibration, the composition of the first-order frequency is much higher than the second-order frequency; that is, the energy ratio is large, indicating that the sandwich transducer works under the first-order inherent frequency.

**Figure 12:**
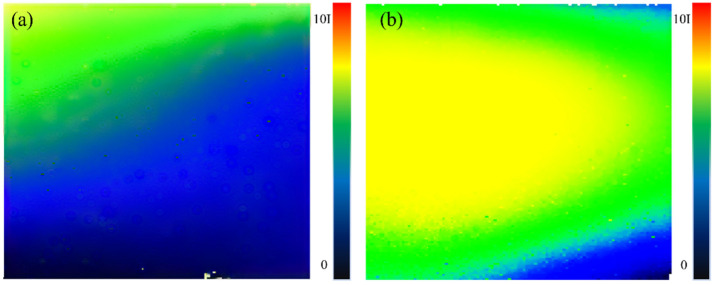
Radial radiation intensity distribution cloud diagram of sandwich transducer: (**a**) vibration intensity cloud diagram of transducer shaft side first order natural frequency; (**b**) Vibration intensity cloud diagram of transducer shaft side under second order natural frequency.

It can be seen from Figure 11a that the first-order radiation intensity distribution is relatively uniform, indicating that the vibration amplitude of the first-order end face particles is basically the same, indicating that there is no vibration mode other than longitudinal vibration, or that the proportion of other vibration modes is very small. Figure 11b shows that the second-order sound field distribution is not uniform. It can be seen that the surface of the transducer is not equal in terms of amplitude vibration, and the radiation intensity distribution is different from the center, indicating that the structural coupling between the piezoelectric ceramic, the front and rear cover plates, and the preload bolt is not uniform, resulting in the overall vibration of the transducer not only along the axial vibration but also in the radial vibration mode. The ideal vibration state of the transducer is that in which there is only longitudinal vibration, that is, there is no radial vibration of the transducer, which does not include the small side vibrations caused by material deformation. When there is radial vibration, that is, when there is vibration along the normal direction of the cylinder, it will have a super positional effect on the longitudinal vibration. Therefore, a radiation intensity scanning experiment was carried out on the cylinder of the transducer. The scanning area is a quarter of the circumferential area of the front mass block. The scanning results are shown in Figure 12.

Compared with the end face’s radiation intensity, the radial radiation intensity is very small, indicating that the radial vibration amplitude is relatively small. Figure 12a is the first-order side radiation intensity distribution. Although it is unevenly distributed along the circumferential direction, considering that its value is very low, the influence of bending vibration on the end face radiation intensity distribution can be ignored, which is consistent with the measurement results in Figure 11a. Figure 12b shows a uniform distribution along the circumferential direction and a uniform variation along the height direction, which is consistent with the radial vibration caused by radial deformation due to longitudinal tensile and compressive deformation.

## 6. Conclusions

In this paper, a natural frequency calculation model of a sandwich transducer is established based on continuum mechanics and a one-dimensional wave equation. Considering the influence of the preload bolt on the overall response frequency of the transducer, the equivalent elastic model-based quantitative method is proposed to calculate the equivalent sound velocity and elastic modulus of each part of the transducer. Based on the half-wavelength theory, the mathematical relationship between the natural frequency of the transducer and the length of each equivalent component is obtained. According to the one-dimensional simplified model proposed in this study, a sandwich transducer with a natural frequency of 15 KHz was designed and fabricated. The vibration signal of the end face of the transducer was collected by a laser vibrometer for spectrum analysis. The accuracy of the one-dimensional vibration model of the transducer was verified, and the effects of different voltages, operating frequencies, and signal continuity on the working state of the transducer were analyzed. Finally, a transducer end vibration-scanning system was built to evaluate the working state of the transducer, and the vibration state of the first-order and second-order end surfaces and the radial surface of the transducer were obtained. The results show that the axial vibration response of the transducer at the first-order resonant frequency is much larger than the radial vibration response. This study not only simplifies the calculation of the natural frequency of the transducer but also provides a reference for the application of the power transducer in the field of ultrasonic machining. This study plays a guiding role in quantifying the working state of the transducer and adjusting the process parameters of ultrasonic machining.

## Figures and Tables

**Table 1:** Boundary conditions of transducer vibration node’s sections.

Cross-Sectional Position	Boundary Conditions
The right side of the cross-section	$v_{1}(0)=0$
	$v_{1}(L_{1})=v_{2}(0)$
	$v_{1}(L_{2})=v_{f}$
	$F_{1}(L_{1})=F_{2}(0)$
	$F_{2}(L_{2})=-Z_{f}v_{f}$
The left side of the cross-section	$v_{3}(L_{3})=0$
	$v_{3}(0)=v_{4}(L_{4})$
	$v_{4}(0)=v_{b}$
	$F_{3}(0)=F_{4}(L_{4})$
	$F_{4}(0)=0$

**Table 2:** Parameters of each component material of the transducer.

Materials	Velocity of Sound m/s	Density kg/m^3^	Elastic Modulus Gpa	Poisson’s Ratio	Elastic Constants S^D^_33_ 10^10^ m^2^/N	Piezoelectric Voltage Constants g_33_ 10^−3^ V·m/N
PZT-8	3100	7600	72	-	8.5	25.4
duralumin	5200	2790	72	0.34	-	-
C45E4 steel	5170	7850	200	0.28	-	-

**Table 3:** Equivalent parameters of each component material of the transducer.

Structure of the Transducer	Velocity of Sound c (m/s)	Density ρ (Kg/m^3^)	Elastic Modulus *E* (Gpa)
Piezoelectric Ceramics PZT-8	3367	7632	88.52
Aluminum front mass block	5019	3272	72
Aluminum back mass block	5019	3272	72

## Data Availability

Not applicable.
